# P-1400. Feasibility, Acceptability and Impact of Self-Sample Collection for Extra-genital STI Testing: Experience from a High Complexity VA Facility

**DOI:** 10.1093/ofid/ofae631.1575

**Published:** 2025-01-29

**Authors:** Rachel Waitzman, Amy Shumaker, Kristen A Allen, Kaylee Bray, Michelle Mercurio, Stephanie Walker, Maria Navas, Lewis S Musoke, Puja Van Epps

**Affiliations:** Oregon Health & Science University, Portland, Oregon; VA Northeast Ohio Healthcare System, Cleveland, Ohio; VA Northeast Ohio Health Care System, Westlake, Ohio; VA Northeast Ohio Healthcare System, Cleveland, Ohio; VA Northeast Ohio Healthcare System, Cleveland, Ohio; VA Northeast Ohio Healthcare System, Cleveland, Ohio; VA Northeast Ohio Healthcare System, Cleveland, Ohio; Veterans Affairs Northeast Ohio Healthcare System, Cleveland, OH; Veterans Health Administration, Case Western Reserve University School of Medicine, Cleveland, Ohio

## Abstract

**Background:**

Routine screening of all exposed extra-genital sites for sexually transmitted infections (STIs), specifically Neisseria gonorrhoeae (GC) and Chlamydia trachomatis (CT), is recommended for individuals at high risk, including some men who have sex with men (MSM) and individuals receiving Pre-Exposure Prophylaxis (PrEP). However, there are a number of barriers to screening including privacy and provider availability. While self-collected extra-genital STI samples have the potential to mitigate some of these barriers, this approach has not yet achieved widespread use. This study evaluates the implementation of self-collection of extra-genital STI tests at the VA Northeast Ohio Healthcare System (VANEOHS), assesses acceptability and measures impact on testing rates.

**Figure:**
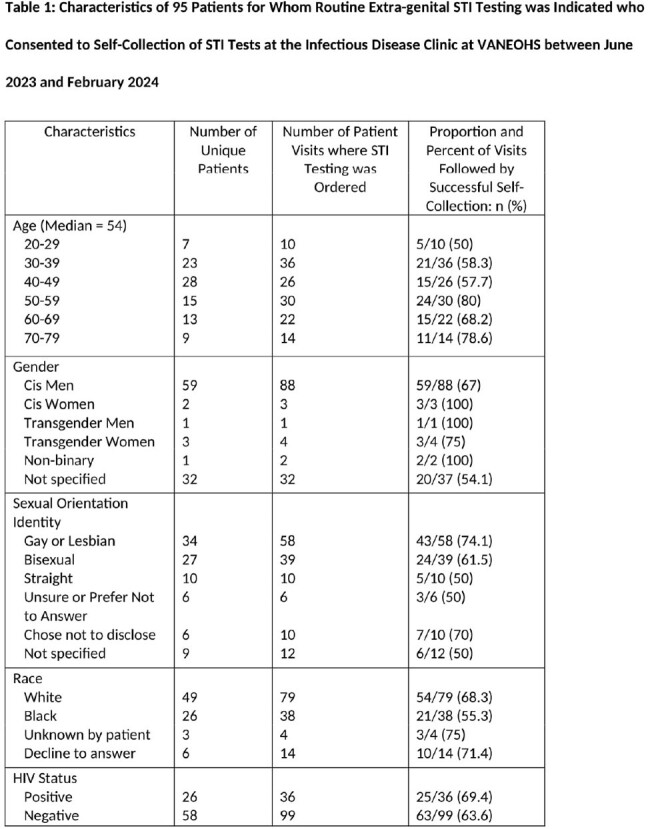

**Methods:**

The study included people with HIV (PWH) and patients on PrEP at the VANEOHS’s infectious diseases clinic and covered 10 Ohio counties. Patients chose between self- and provider-collection. We followed GC and CT rectal and pharyngeal testing frequency and test completion rates and characterized reasons for unsuccessful completion. We also compared extra-genital GC/CT testing rates before and after the availability of self-collection. Finally, we deployed an anonymous survey to assess acceptability of self-collection and ease of use. We used descriptive statistics to report completion rates, survey findings and changes in rates of testing over time.

**Figure:**
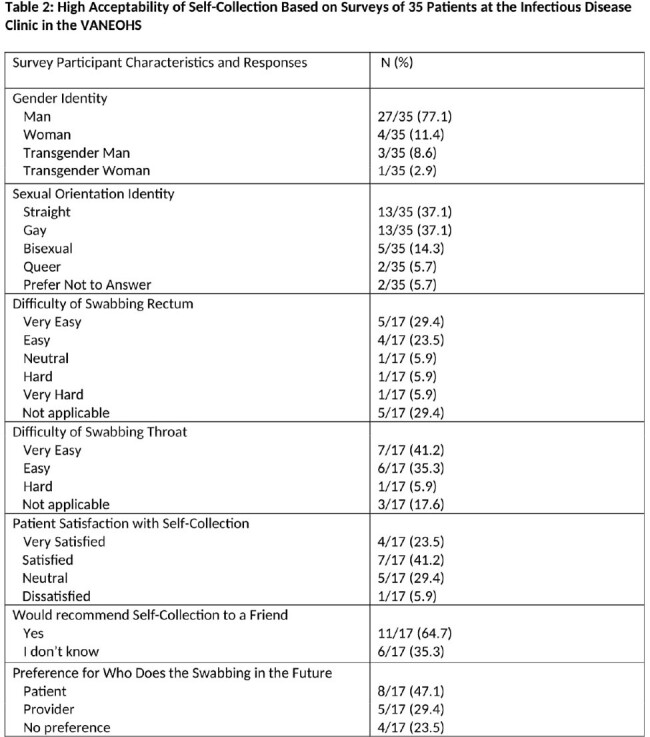

**Results:**

Of the 276 tests ordered for self-collection for 95 unique veterans, 82 pharyngeal and 60 rectal GC/CT rectal tests were successfully completed and interpreted in the first 8 months of the study (Table 1). The testing rate increased from 8 tests/month to 15 tests/month for pharyngeal GC/CT and from 6 tests/month to 10 tests/month for rectal GC/CT (Figure 1). Of the survey respondents who performed self-collection, 11 of 17 were satisfied or very satisfied with self-collection and most found it easy or very easy to swab their own rectum (9/12) and throat (13/14) (Table 2).

**Figure:**
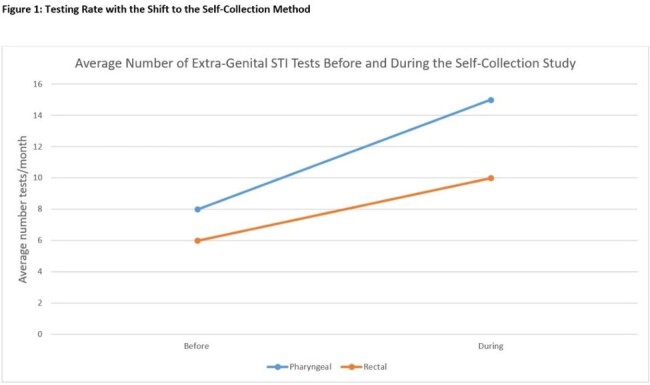

**Conclusion:**

Self-sample collection for extra-genital testing is feasible in a large healthcare network. It is highly desired by patients and has the potential to improve access and improve STI screening rates.

**Disclosures:**

**All Authors**: No reported disclosures

